# Satellite‐Based Long‐Term Spatiotemporal Trends in Ambient NO_2_ Concentrations and Attributable Health Burdens in China From 2005 to 2020

**DOI:** 10.1029/2023GH000798

**Published:** 2023-05-17

**Authors:** Keyong Huang, Qingyang Zhu, Xiangfeng Lu, Dongfeng Gu, Yang Liu

**Affiliations:** ^1^ Department of Epidemiology Fuwai Hospital, National Center for Cardiovascular Diseases Chinese Academy of Medical Sciences and Peking Union Medical College Beijing China; ^2^ Key Laboratory of Cardiovascular Epidemiology Chinese Academy of Medical Sciences Beijing China; ^3^ Gangarosa Department of Environmental Health Rollins School of Public Health Emory University Atlanta GA USA; ^4^ School of Medicine Southern University of Science and Technology Shenzhen China

**Keywords:** air pollution, nitrogen dioxide, spatiotemporal trend, disease burden, China

## Abstract

Despite the recent development of using satellite remote sensing to predict surface NO_2_ levels in China, methods for estimating reliable historical NO_2_ exposure, especially before the establishment of NO_2_ monitoring network in 2013, are still rare. A gap‐filling model was first adopted to impute the missing NO_2_ column densities from satellite, then an ensemble machine learning model incorporating three base learners was developed to estimate the spatiotemporal pattern of monthly mean NO_2_ concentrations at 0.05° spatial resolution from 2005 to 2020 in China. Further, we applied the exposure data set with epidemiologically derived exposure response relations to estimate the annual NO_2_ associated mortality burdens in China. The coverage of satellite NO_2_ column densities increased from 46.9% to 100% after gap‐filling. The ensemble model predictions had good agreement with observations, and the sample‐based, temporal and spatial cross‐validation (CV) *R* ^2^ were 0.88, 0.82, and 0.73, respectively. In addition, our model can provide accurate historical NO_2_ concentrations, with both by‐year CV *R* ^2^ and external separate year validation *R* ^2^ achieving 0.80. The estimated national NO_2_ levels showed a increasing trend during 2005–2011, then decreased gradually until 2020, especially in 2012–2015. The estimated annual mortality burden attributable to long‐term NO_2_ exposure ranged from 305 thousand to 416 thousand, and varied considerably across provinces in China. This satellite‐based ensemble model could provide reliable long‐term NO_2_ predictions at a high spatial resolution with complete coverage for environmental and epidemiological studies in China. Our results also highlighted the heavy disease burden by NO_2_ and call for more targeted policies to reduce the emission of nitrogen oxides in China.

## Key Points

- We developed an ensemble machine learning model on NO_2_ levels using column densities from Ozone Monitoring Instrument satellite as main predictors
- Our model obtained sample‐based, temporal and spatial cross‐validation *R* ^2^ of 0.88, 0.82, and 0.73, respectively
- The estimated annual mortality burden attributable to chronic NO_2_ exposure ranged from 305 thousand to 416 thousand in China

## 1 Introduction

Ambient nitrogen dioxide (NO_2_) is a major air pollutant, mainly originating from traffic and fuel combustion emissions. Several epidemiological studies have found that exposure to ambient NO_2_ was associated with decreased lung function, cardiopulmonary diseases, and premature deaths independent of other air pollutants (S. Huang et al., 2021; Meng et al., 2021; Strassmann et al., 2021). In addition, ambient NO_2_ is a key precursor of a series of secondary pollutants, such as ozone and fine particulate matter (PM_2.5_). In 2021, the World Health Organization (WHO) tightened the air quality guideline, reducing the annul NO_2_ standard level from 40 to 10 μg/m^3^. However, mainly due to the lack of historical surface NO_2_ data before 2013, the long‐term spatiotemporal trend of NO_2_ and its chronic health effects were rarely reported in China (Yin et al., 2020).

With a high spatiotemporal coverage, satellite remote sensing technology has become a promising tool to estimate surface air pollutants, and shown potential to fill the gap left by ground fixed monitors, especially in regions with sparse monitoring (Cooper et al., 2022; Di et al., 2020; K. Huang et al., 2018; Ma et al., 2022). Correspondingly, several statistical models have been developed to convert satellite data to surface air pollutants, such as land use regression, geographically weighted regression and machine learning algorithms (Geddes et al., 2016; C. Huang et al., 2021; Song et al., 2019; Zhan et al., 2018). For example, Geddes et al. (2016) estimated the global NO_2_ concentrations at 10‐km resolution from 1996 to 2012 using NO_2_ tropospheric column densities from satellite instruments. In China, Zhan et al. (2018) developed a hybrid random forest and spatiotemporal kriging model using NO_2_ column densities from satellite Ozone Monitoring Instrument (OMI), and predicted surface NO_2_ levels at 10‐km resolution from 2013 to 2016. Despite the valuable NO_2_ predictions from previous studies, there are still some aspects to be improved to promote epidemiological study and disease burden estimation in China. First, most of existing studies focused on NO_2_ estimation after the establishment of NO_2_ monitoring network in 2013 (C. Huang et al., 2021; Wang et al., 2022; Wei et al., 2023). Reliable historical NO_2_ data before 2013 were still rare. Second, most of the national or regional NO_2_ estimations were conducted at a coarse spatial resolution in China (e.g., 10 km) (Qin et al., 2020; Wu et al., 2021; Xu et al., 2021), while studies having a high spatial resolution were often constrained to a relatively short period (Wei et al., 2022). To the best of our knowledge, no existing study has predicted ambient NO_2_ concentrations in China, simultaneously achieving high spatial resolution, and high spatiotemporal coverage to support large‐scale assessments of chronic NO_2_ exposure related adverse health effects. Second, the cloud cover and bright surfaces usually lead to the non‐random missing of satellite NO_2_ column densities (Li & Wu, 2021). The onset of row anomaly since 2007 further increased the proportion of missing values. This anomaly affects the quality of the level 1B radiance data at all wavelengths for a particular viewing direction of OMI (Schenkeveld et al., 2017). Without considering the non‐random missing values, it may lead to exposure misclassification and bias the health effects of NO_2_ exposure in epidemiological studies.

In the current study, we aimed to develop an ensemble machine‐learning model integrating random forest, extreme gradient boosting (XGBoost), and Gradient boosting machine (GBM) algorithms to assess the monthly NO_2_ levels at 0.05° × 0.05° spatial resolution from 2005 to 2020, and to evaluate mortality burden attributable to NO_2_ exposure at the provincial level in China. We first developed a gap filling approach to impute the missing OMI NO_2_ column densities using meteorology, cloud cover, and Copernicus Atmosphere Monitoring Service (CAMS) nitrogen oxides assimilation results. Based on the gap‐filled OMI data, we then trained three separate machine learning models and combined them by a generalized additive model (GAM). We finally estimated the mortality burden related to NO_2_ in each province of China based on the satellite‐derived high resolution NO_2_ data set and the epidemiologically derived exposure response relations.

## 2 Materials and Methods

### 2.1 Study Area

**Figure 1:**
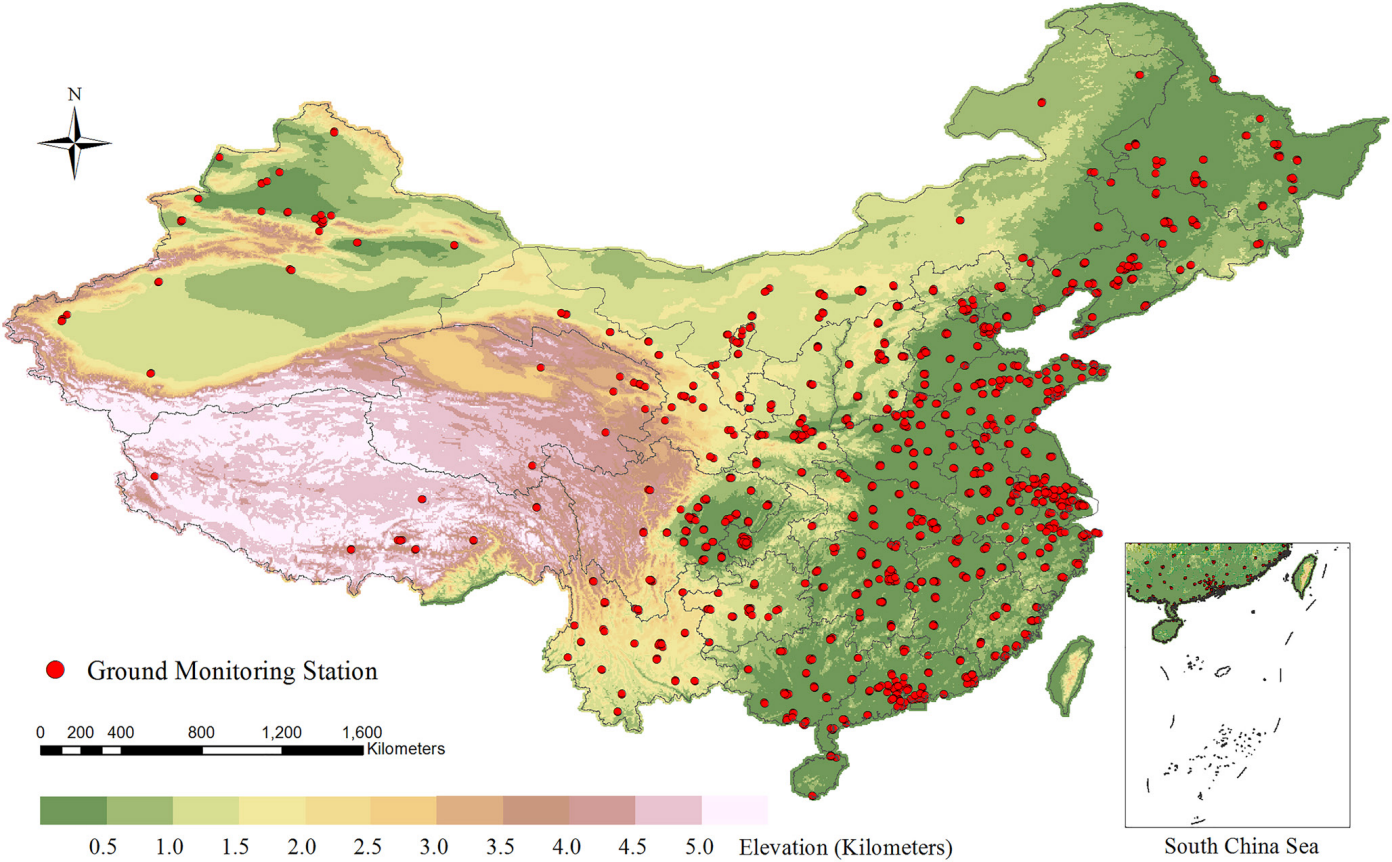
Study domain plus 50‐km buffer and distributions of air quality monitoring stations. Air quality monitors are shown as red dots.

Our study domain covered mainland China, Hong Kong, Macao, and Taiwan (Figure 1). To ensure prediction accuracy at the border area, a 50‐km buffer region was created around the national boundary. We constructed a 0.05° resolution modeling grid over this study domain for data integration and model training, totaling 399,513 grid cells.

### 2.2 Ground NO_2_ Measurements

Ground‐level hourly NO_2_ measurements were collected from ∼1,500 air quality monitoring stations administered by the China National Environmental Monitoring Center (http://www.cnemc.cn/) (Figure 1). We removed repeated identical NO_2_ values for at least eight continuous hours because these measurements were likely caused by instrument malfunction. Days with less than 18 (75%) hourly measurements were excluded. In addition, those months with less than 20 days of valid NO_2_ measurements were also removed. We multiplied the NO_2_ values before September 2018 by 0.92, since the monitoring condition was amended from standard atmospheric state (273 K, 101.325 kPa) to reference state (298.15 K, 101.325 kPa) for gaseous pollutants (Wu et al., 2021). Finally, daily mean NO_2_ concentrations from each station were aggregated to the monthly level. We used the data during 2014–2019 for model training and data during 2020 for external validation.

### 2.3 Satellite Retrieved NO_2_ Data

We obtained the satellite retrieved tropospheric vertical column density (VCD) of NO_2_ during 2005–2020 from OMI NO_2_ level‐3 data product (OMNO2d, 0.25° × 0.25° resolution) (Krotkov et al., 2017). The OMI instrument onboard the Aura satellite is mainly used to observe the ozone profile, air quality and climate change, with a nearly global coverage on a daily basis since July 2004. To ensure the data quality, we removed those pixels with a cloud fraction >30%. In the current study, we first imputed the missing NO_2_ VCD, then interpolated it to the 0.05° resolution grid by inverse distance weighting (IDW).

### 2.4 Meteorological and Land Use Data

Meteorological parameters in 2005–2020 were obtained from the fifth generation European Center for Medium‐Range Weather Forecasts atmospheric re‐analysis (ERA5) (Hersbach et al., 2020). It has been shown that meteorological conditions play crucial roles in formatting ambient NO_2_ levels. We included air temperature, relative humidity, wind speed, planetary boundary layer height, total precipitation, surface solar radiation and thermal radiation, etc. as predictors in this study. It has a spatial resolution of 0.25° × 0.25°, and were downscaled to 0.05° grid by IDW method.

We downloaded annual land cover maps at 300 m resolution from the European Space Agency Climate Change Initiative for 2005–2015 (https://vest.agrisemantics.org/content/land-cover-cci-product-user-guide) and the Copernicus Climate Change Service Climate Data Store (CDS) for 2016–2020 (https://cds.climate.copernicus.eu/cdsapp#!/dataset/satellite-land-cover?tab=overview). From these two products, we extracted the area of urban cover, forest cover, shrub, grass, wetland, cropland, water body and bare land in each grid cell. In addition, we calculated the highway versus non‐highway lengths in each grid based on the Global Roads Open Access Data Set (https://sedac.ciesin.columbia.edu/data/set/groads-global-roads-open-access-v1).

### 2.5 Other Predictors

Additional predictors were used in this study to improve the NO_2_ model prediction accuracy, including the lightning flash density, simulations from the Modern‐Era Retrospective analysis for Research and Applications Version 2 (MERRA‐2), monthly total emissions of nitrogen oxides, elevation and population density.

Lightning flash is an important contributor to the formation of NOx, and we obtained monthly lightning flash density data from the Harvard‐NASA Emissions Component at 0.5° × 0.625° resolution (Murray et al., 2012). MERRA‐2 is the latest NASA atmospheric reanalysis at 0.5° × 0.625° resolution, and monthly sulfate ion, organic carbon, black carbon, dust and seasalt simulations were extracted (Gelaro et al., 2017). Monthly inventories of total emissions of nitrogen oxides at 0.1° × 0.1° resolution were obtained from CAMS (https://ads.atmosphere.copernicus.eu/cdsapp#!/home). Annual population density data from 2005 to 2020 were obtained from the Oak Ridge National Laboratory at 1‐km resolution (https://landscan.ornl.gov/). We extracted the elevation data at 30‐m resolution from the Advanced Spaceborne Thermal Emission and Reflection Radiometer Global Digital Elevation Model, version 3.

### 2.6 Satellite VCD of NO_2_ Gap‐Filling Model

In this study, we employed a random forest model, including CAMS total column nitrogen dioxide, total column nitrogen monoxide, cloud fraction, air temperature, dew point temperature, elevation and spatiotemporal trends, to impute the missing OMI VCD values. To consider the temporal trends of OMI VCD, a rolling 3‐day sampling window was used to impute the missing OMI VCD values on the middle day. There are two main hyperparameters in the random forest model, that is, the number of predictors selected for each split (mtry) and the number of trees grown in the forest (ntree). After comparing the performance of different settings, we set mtry and ntree as 5 and 500, respectively.

### 2.7 Ground Level NO_2_ Prediction Model

We used an ensemble model to estimate monthly mean ambient NO_2_ concentrations in China. We first trained three independent machine learning algorithms, including random forest, XGBoost, and GBM. According to Wu et al. (2021), we used the scaling factors (i.e., ratios of the surface NO_2_ concentrations to the OMI VCD) as the dependent variable rather than the surface NO_2_ concentrations. Then, we multiplied the scaling factors and OMI VCD to obtain the ground level NO_2_ values. The scaling factors measure the vertical proportion of NO_2_, and have been reported to improve the prediction accuracy of historical NO_2_ levels (Wu et al., 2021). Finally, we combined the NO_2_ predictions from three individual machine learning models by GAM into an ensemble model. Comprehensively considering the training efficiency and model performance, we set the key parameters as the following: the number of trees was 500 for random forest, XGBoost, and GBM. The number of variables per split was 15 for random forest. The maximum tree depth was 10 for XGBoost and GBM.

We conducted sample‐based 10‐fold cross validation (CV) to evaluate the model performance. Correspondingly, the entire data set was randomly split into 10 groups, with each group containing 10% of the data. In each round of CV, nine groups of data were selected to fit the model, which was then used to predict on the remaining group. This process was repeated 10 times until every group was predicted. In addition, we also performed the 10‐fold spatial and temporal CV to evaluate the model prediction accuracy at unmonitored site and time. For spatial CV, we randomly selected 90% of the locations to fit the model and made predictions on the remaining locations. Similarly, for temporal CV, we selected 90% of the months to fit the model and made predictions on the remaining months. Additionally, we conducted by‐year CV to evaluate the model's hindcast performance, in which 1 year was used for testing and remaining years for training. Furthermore, a separate time period, January 2020–December 2020, was used to characterize the hindcast prediction error. Statistical indicators, such as coefficient of determination (*R* ^2^) and root mean squared prediction error (RMSE), were calculated to evaluate the model performance.

### 2.8 Mortality Burden Attributable to NO_2_ Exposure

We calculated the mortality burden caused by NO_2_ exposure at provincial level from 2005 to 2020 in China. The annual provincial population and mortality data from 2005 to 2019 were downloaded from the China Statistical Year Book (http://www.stats.gov.cn/). Since the data were still not available for 2020, we used the population and mortality data in 2019 instead. The attributable deaths were calculated using the following equation.

$${RR}_{C}{\;=\;RR}^{\frac{\left({NO}_{2}-Ref\right)}{10}}$$

$${AD}_{ij}=\left({RR}_{C}-1\right)/{RR}_{C}\times {Pop}_{ij}\times I_{ij}$$

where RR_*C*_ is the relative risk for all cause mortality related to NO_2_ exposure above the reference value. Recommended by the recent WHO air quality guidelines, we used the RR value of 1.02 (95% CI: 1.01–1.04) for all cause mortality related to per 10 μg/m^3^ increase in NO_2_ (Huangfu & Atkinson, 2020). Since no obvious threshold NO_2_ value was reported in previous epidemiological studies, we used the counterfactual zero level as the reference (Meng et al., 2021). AD_ij_ is the attributed deaths in province *i* at year *j*. Pop_*ij*_ and *I*_*ij*_ is the total population and baseline mortality rate in province *i* at year *j*. In addition, to eliminate the motality burden attributable to population growth, the annual NO_2_ related deaths were recalculated using the population and mortality data in 2005 as the reference.

## 3 Results

### 3.1 Descriptive Statistics

The average ground NO_2_ concentrations during 2014–2019 was 30.4 μg/m^3^, with standard deviation of 14.6 μg/m^3^. The annual mean NO_2_ levels decreased by 7 μg/m^3^ from 2014 to 2015, and then keep relatively constant from 2015 to 2019 (Table S1 in Supporting Information S1). The NO_2_ levels in China were much higher than the annual NO_2_ limit (10 μg/m^3^) of WHO air quality guidelines.

### 3.2 OMI VCD Gap‐Filling by Random Forest

The mean coverage of OMI VCD of NO_2_ in China from 2005 to 2019 was 46.9%. The north of China (∼65%) has a higher coverage than the south (<35%), especially in the southwest (Table S2 and Figure S1 in Supporting Information S1). After imputation, the coverage of OMI VCD increased to 100%. The daily gap filling model achieved an average out‐of‐bag *R* ^2^ of 0.91, with interquartile ranges from 0.88 to 0.94. The spatial distribution of OMI VCD after imputation was almost consistent with that before imputation (Figure S2 in Supporting Information S1), with much higher values observed in north and east of China. The average OMI VCD increased after gap filling (Table S2 in Supporting Information S1), possibly because areas where NO_2_ is more often missing are more polluted (Figures S1 and S2 in Supporting Information S1).

### 3.3 Ground NO_2_ Prediction Model Performance

The validation results of the three separate machine learning models and ensemble model were shown in Table 1. Among the individual machine learning models, the XGboost had the highest CV *R* ^2^ and the lowest CV RMSE, followed by GBM. The ensemble model outperformed three individual machine learners (*R* ^2^, random forest: 0.85, XGboost: 0.87, GBM: 0.87). The sample‐based CV *R* ^2^ and RMSE of the ensemble model for monthly NO_2_ were 0.88 and 5.14 μg/m^3^, respectively, implying a relatively good agreement between model predictions and ground measurements. The temporal CV had a slightly lower *R* ^2^ of 0.82 and a higher RMSE of 6.14 μg/m^3^. The spatial CV had a less satisfying model performance with a *R* ^2^ of 0.73 and a RMSE of 7.57 μg/m^3^.

**Table 1:** Model Performance for Individual Machine Learning Model and Ensemble Model

*R* ^2^ (RMSE, μg/m^3^)	Individual model			Ensemble model
	Random forest	XGboost	GBM	
Sample‐based CV	0.85 (5.74)	0.87 (5.20)	0.87 (5.36)	0.88 (5.14)
Temporal CV	0.78 (6.85)	0.82 (6.27)	0.81 (6.32)	0.82 (6.14)
Spatial CV	0.75 (7.36)	0.72 (7.70)	0.72 (7.69)	0.73 (7.57)

*Note.* RMSE, root mean squared prediction error; XGBoost, extreme gradient boosting; GBM, Gradient boosting machine; CV, cross validation.

**Figure 2:**
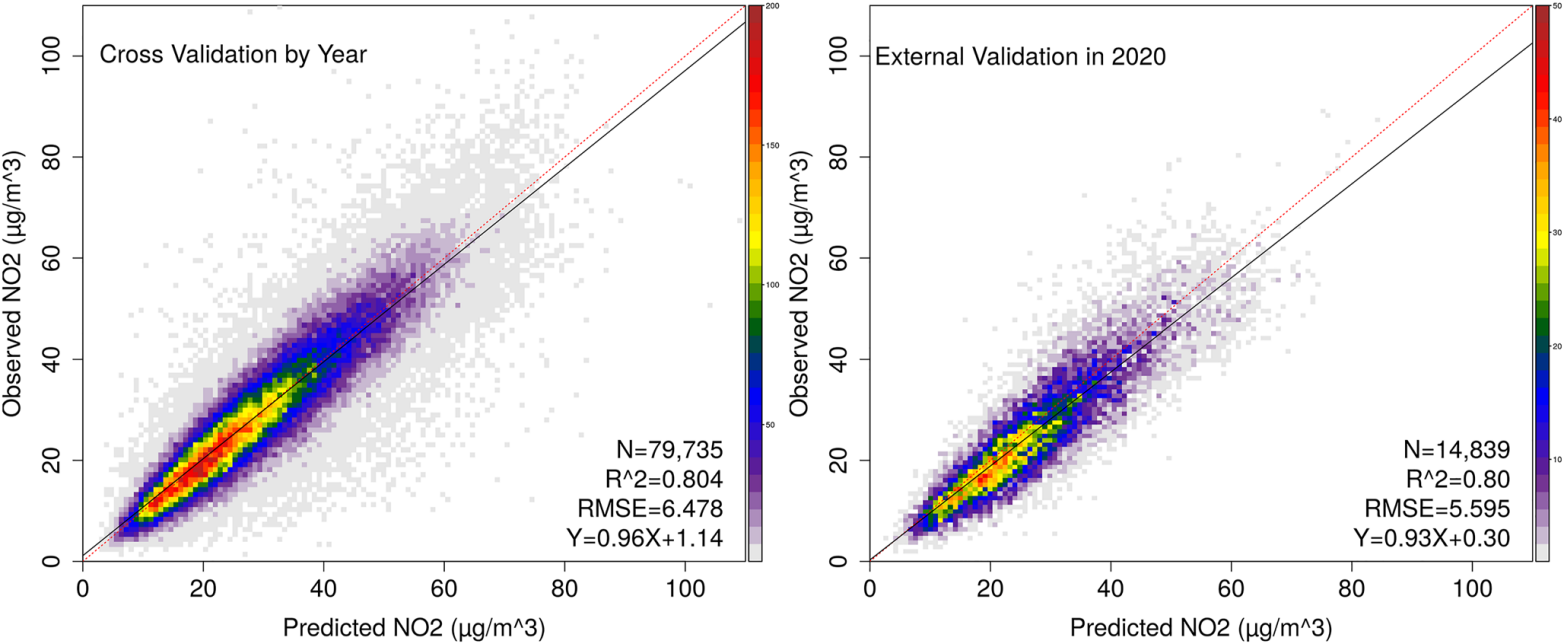
By‐year cross validation and external validation using data from year 2020.

To further evaluate the model's hindcast capability, we conducted by‐year CV and external validation using data from January to December 2020 (Figure 2). In the by‐year CV, the ensemble model predictions matched well with ground observations, with a *R* ^2^ of 0.80 and a RMSE of 6.48 μg/m^3^. In the external validation, the results shown that our ensemble model fitted by data of 2014–2019 can predict NO_2_ levels in 2020 with high accuracy (*R* ^2^ = 0.80, RMSE = 5.60 μg/m^3^).

### 3.4 Spatial and Temporal Distribution of NO_2_

Based on the ensemble model, we obtained monthly ground NO_2_ levels in China from 2005 to 2020. We estimated that almost entire population (99.4%) of China lived in areas where NO_2_ levels exceeded the 2021 WHO air quality guideline, set at 10 μg/m^3^, with 18.3% (255.6 million people) exceeding the WHO interim targets 1 (40 μg/m^3^). For the main economic and population concentrated areas, the proportions exceeding 40 μg/m^3^ in Beijing‐Tianjin‐Hebei (BTH) area, Yangtze River Delta (YRD), Pearl River Delta (PRD) and Fenwei Plain (FWP) were 71.6%, 21.7%, 45.8%, and 29.9%, respectively.

**Figure 3:**
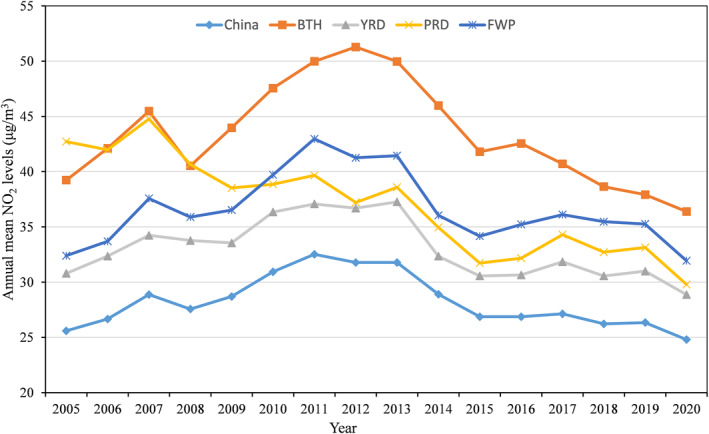
National and sub‐regional annual population weighted NO_2_ concentrations from 2005 to 2020. BTH, Beijing‐Tianjin‐Hebei area; YRD, Yangtze River Delta; PRD, Pearl River Delta; FWP, Fenwei Plain.

Figure 3 displayed the time series of annual population weighted NO_2_ for China and four subregions. The national population weighted NO_2_ levels began to increase from 2005 to 2007, and experienced the first decrease in 2008. Then it continued to increase until its highest level in 2011, reaching 32.5 μg/m^3^. After 2011, it decreased gradually until 2020, especially in 2012–2015. When stratified by region, NO_2_ levels in BTH, YRD, PRD, and FWP were all higher than the national average, with the highest observed in BTH. Similar to the national trend, the NO_2_ in BTH, YRD, and FWP reached the peak around 2011–2012, then decrease until 2020. However, the NO_2_ in PRD generally shown a continuous downward trend in our study period. During the lock down period due to Covid‐19, we observed a significant decrease of NO_2_ concentrations. Compared with the data of the same period in 2016–2019, the NO_2_ levels in China and Wuhan city decreased by 16.1% and 28.8% from January to April 2020 in lock down (Figure S3 in Supporting Information S1).

**Figure 4:**
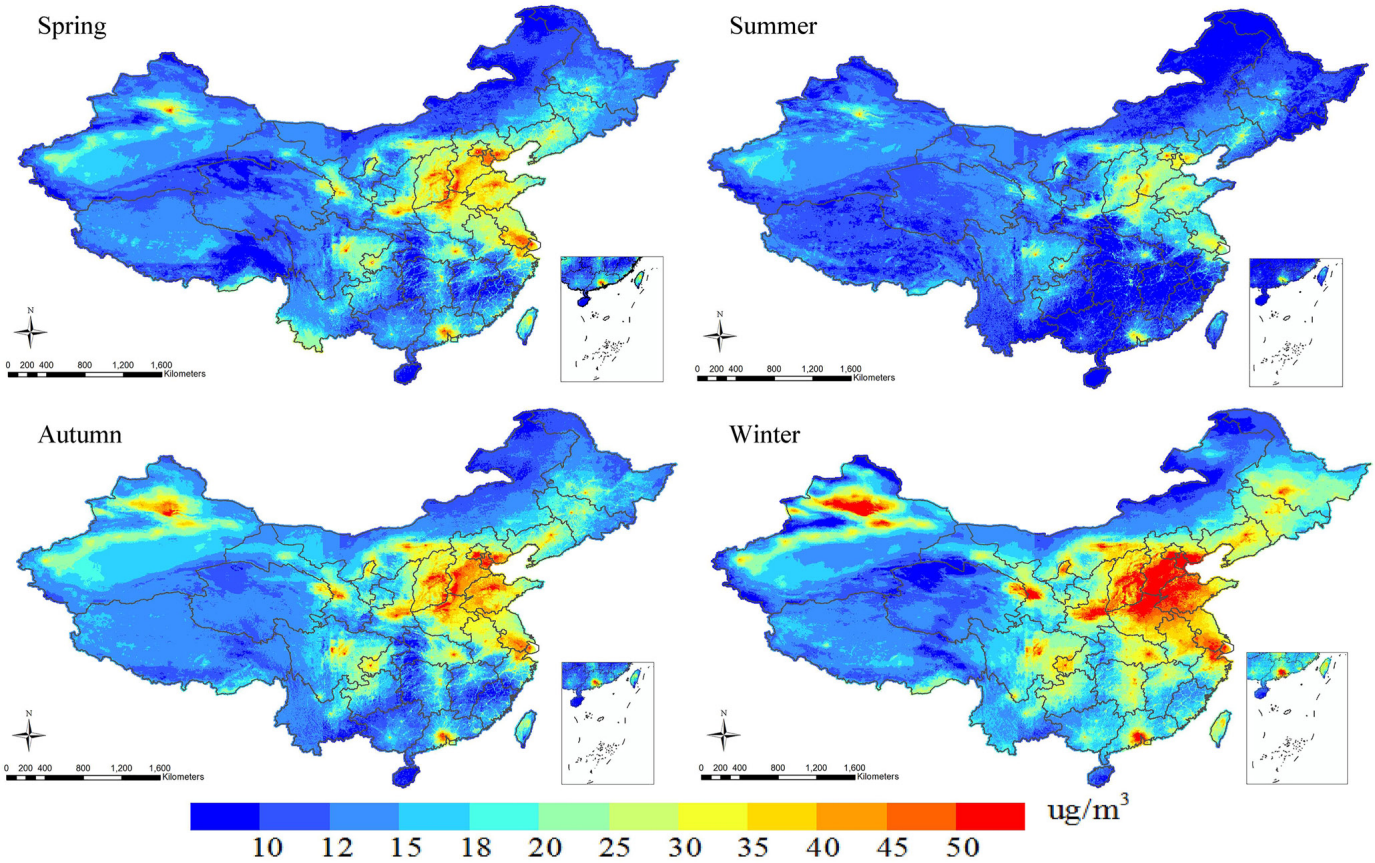
Spatial distributions of seasonal ambient NO_2_ concentrations in China from 2005 to 2020.

The spatial distribution of NO_2_ levels in China by season were shown in Figure 4. The NO_2_ levels peaked in winter and were the lowest in summer. The population weighted NO_2_ concentrations in China were predicted to be 27.4, 19.6, 29.6, and 36.3 μg/m^3^ in spring, summer, autumn and winter, respectively. Spatial trends of NO_2_ levels over China were comparable in four seasons, with relatively higher pollution in Beijing, Tianjin, southern Hebei, and northern Henan. Other NO_2_ hot spots included the YRD, and the PRD.

### 3.5 Mortality Burden Attributable to Ambient NO_2_

**Figure 5:**
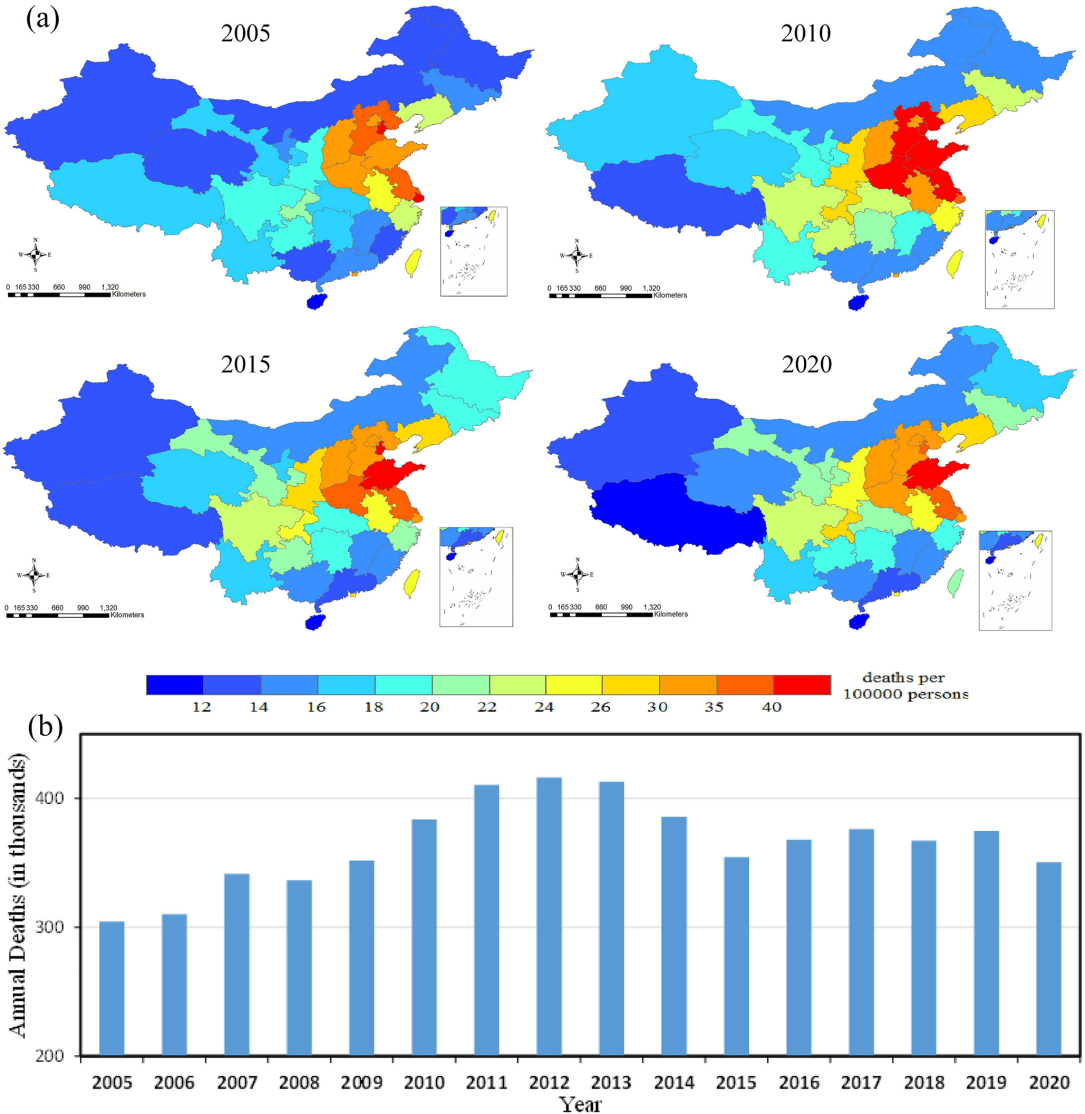
Annual mean mortality burden attributable to long‐term NO_2_ exposure in China. (a), annual NO_2_ related mortality burden per 100,000 persons at provincial level in 2005, 2010, 2015, and 2020. (b), national mean NO_2_ related mortality burden from 2005 to 2020.

As shown in Figure 5, the annual mortality burden attributable to NO_2_ exposure in China ranged from 305 thousand (2005) to 416 thousand (2012). Overall, it shows a trend of rising first and then declining before 2015, then keeping relatively stable during 2016–2019, followed by a reduction in 2020. If we further subtract the disease burden attributable to population growth by using the population data in 2005, we can still observe a similar trend across the years. The per‐capita deaths were higher in eastern China, especially in Tianjin, Shandong, Jiangsu, Hebei and Shanghai (39.1–48.1 per 100,000 persons), and lower in Hainan, Tibet, and Xinjiang (10.8–14.5 per 100,000 persons). We also calculated the provincial absolute number of deaths caused by ambient NO_2_ pollution from 2005 to 2020, and found that the provinces with higher 16‐year total NO_2_ related mortality burden included Shandong, Henan, and Jiangsu province (538 thousand to 668 thousand), and lower in Macao, Tibet, and Qinghai (2 thousand to 14 thousand).

## 4 Discussion

In the current study, we filled the research gap by estimating the 16‐year spatiotemporal trends in NO_2_ and associated mortality burden across provinces in China. We first produced a high spatial resolution (0.05° × 0.05°) and long‐term (2005–2020) data sets of surface NO_2_ concentrations, which enable us to resolve NO_2_ variations at small scale. In addition, we found almost entire population of China lived in regions exceeding 2021 WHO guideline annual average NO_2_ levels (10 μg/m^3^), resulting in 305 thousand to 416 thousand deaths annually from 2005 to 2020.

Accurately estimating the NO_2_ concentrations is a crucial step toward epidemiological studies and disease burden estimation. In this study, we developed a high performance ensemble model to predict surface NO_2_ levels, and obtained a relatively high prediction accuracy (sample‐based, temporal, and spatial CV *R* ^2^: 0.877, 0.824, and 0.732), which was better than most existing models (Chi et al., 2021; Z. Huang et al., 2022; Qin et al., 2017; Zhan et al., 2018). Our model has several advantages over previous studies. First, in contrast to most previous studies which trained a single algorithm (Z. Huang et al., 2022; Zhan et al., 2018), we ensembled multiple machine learners by GAM algorithm and obtained better model performance compared to the individual learner. Second, our model exhibited a good capability of predicting historical NO_2_ estimates at high spatial resolution. Due to lack of routine NO_2_ monitoring data before 2013 in China, it's essential to develop NO_2_ prediction models which can provide accurate historical exposure estimates. However, most existing studies focused on a limited time period and ignored the model's capability of predicting historical NO_2_ concentrations, especially the NO_2_ levels before 2013. Only a few studies have evaluated their model's hindcast performance, showing unsatisfying accuracy. For example, C. Huang et al. (2021) estimated daily NO_2_ exposure during 2013–2019 in China using an ensemble model, and obtained a CV *R* ^2^ of 0.72 and a RMSE of 10.61 μg/m^3^. However, the model's accuracy decreased when predicting historical NO_2_ concentrations (by‐year CV *R* ^2^: 0.68, RMSE:11.43 μg/m^3^). In addition, atmospheric NO_2_ mainly comes from traffic and industrial emissions and has a short lifetime, likely forming local pollution hotspots. Thus, previous models with coarse spatial resolution may not be able to resolve the NO_2_ variations at small scale, leading to bias in exposure assessment. In the current study, our ensemble model displayed a high accuracy of predicting historical NO_2_ levels at 0.05° × 0.05° resolution, obtaining by‐year CV *R* ^2^ of 0.80 (RMSE: 6.5 μg/m^3^) and a separate time (year 2020) validation *R* ^2^ of 0.80 (RMSE: 5.6 μg/m^3^). Third, we proposed a gap filling method to impute the missing OMI VCD values and achieved a 100% spatiotemporal coverage in NO_2_ estimation. Some studies have tried the linear regression, mixed model, or temporal convolution methods to impute the missing OMI VCD values, but obtained limited prediction accuracy and the generalizability is uncertain (de Hoogh et al., 2019; Li & Wu, 2021; Wu et al., 2021). For instance, de Hoogh et al. (2019) used a liner mixed effect model to impute the missing OMI data and obtained CV *R* ^2^ of 0.68. He et al. (2020) filled the satellite data from 2015 to 2018 in China by combining the OMI and Global Ozone Monitoring Experiment‐2B (GOME‐2B) measurements using machine learning and an adaptive weighted temporal fitting method. They increased the OMI NO_2_ coverage by 40%, and obtained a cross‐validation *R* ^2^ of 0.89. However, their final gap‐filled OMI NO_2_ did not reach the complete spatial coverage. Li and Wu (2021) imputed the missing OMI NO_2_ data at 1 × 1 km resolution in only 1 year (2015) using the full residual deep learning method. They obtained a high validation *R* ^2^ of 0.98, but the accuracy of long‐term imputation was uncertain. In our study, we built a random forest gap filling model to impute OMI NO_2_ data incorporating several publicly available covariates, and obtained a 100% complete spatial coverage and a satisfactory validation *R* ^2^ of 0.91, which will greatly reduce the bias in NO_2_ exposure and related health impact assessment.

We observed a first increase then decrease trend of NO_2_ concentrations from 2005 to 2020 in China, which was consistent with trend of NOx emissions in China (Jiang et al., 2020). The rapid growth of industrial and vehicle NOx emissions lead to the increasing trend of NO_2_ levels during 2005–2011. After 2011, the NO_2_ levels showed a decreasing trend, especially in 2012–2015. The declined trend during this period was probably due to environmental protection policies in China. In 2011–2015, the Chinese government initiated the 12th Five‐Year‐Plan (FYP) and set a stringent target to reduce the NOx emissions (Jiang et al., 2020). Through increasing the use of clean energy and installing denitrification facilities, China has successfully reduced the NOx emissions by 18.6% in 2011–2015. After 2015, the downward trend of NO_2_ concentrations in China tend to be flat, which may be contributed by the vehicle NOx emissions from sharp increase of private cars (Jiang et al., 2020). Similar with recent findings (Cooper et al., 2022), our ensemble model also observed a significant reduction of NO_2_ levels during Covid‐19 lock down period in Wuhan. It demonstrated the impact of emission reduction policy on NO_2_ pollution.

A series of studies have reported the health burden attributable to air pollution in China, but most of them focused on PM_2.5_ and ozone (Cohen et al., 2017; Liang et al., 2020; Yin et al., 2020). As one of the major NOx emission countries world, China has experienced serious air pollution in recent years. However, very few studies have assessed the NO_2_ related disease burden in China (Xue et al., 2023; Zhao et al., 2020). Based on sparse monitoring data in single year of 2016, Zhao et al. estimated 388.5 thousand deaths caused by NO_2_ exposure above 5 μg/m^3^ in China (Zhao et al., 2020). However, the fixed monitors tend to have higher NO_2_ levels, since they are mainly located in eastern urban areas. In addition, the Chinese government has implemented several policies to mitigate the air pollution in the last decades (Lu et al., 2020). Our study found that NO_2_ pollution is also an important risk factor for mortality burden in China, resulting in 305 thousand to 416 thousand annual deaths from 2005 to 2020. And the mortality burden caused by NO_2_ has dramatically declined since the 12th FYP implementation (2011–2015), demonstrating the air pollution controlling efficacy. However, this trend was not evident since 2016, possibly due to fasting growing vehicles (Jiang et al., 2020). Considering the non‐negligible mortality burden by NO_2_, targeted policies focusing on vehicle emission control should be strengthened in China.

Our study has some limitations. First, the spatial resolution of our model can be further increased by using Tropospheric Monitoring Instrument (TROPOMI) satellite retrievals at 3.5 × 7.0 km resolution (Veefkind et al., 2012). However, TROPOMI was launched in October 2017, thus could not meet the needs of long‐term health effects epidemiological studies and health impact assessment. Second, although the exposure response relations between NO_2_ and mortality we used was from a recent large‐scale meta‐analysis adopted by the 2021 WHO air quality guideline (Huangfu & Atkinson, 2020), it mainly relied on studies from the USA and Europe, which may bias the disease burden estimates. More studies on the chronc health effects of NO_2_ from China and other low and middle income countries are still needed. Third, limited by the availability of detailed spatially resolved annual population mortality data in China, we only calculated the NO_2_ related mortality burden at the provincial level. Nonetheless, finer scale estimation of disease burden attributed to NO_2_ exposure may capture more within‐city variations and can be used for further environmental inequality analysis by geographic and socioeconomic factors (Xue et al., 2023).

## 5 Conclusions

In the current study, we presented an ensemble machine learning model to estimate long‐term NO_2_ concentrations at high spatial resolution in China. Based on this model, we produced reliable historical monthly mean NO_2_ estimations at 0.05° resolution, which will greatly enhance epidemiological studies and health impact assessment of chronic NO_2_ exposure in China. Furthermore, our results also indicated that exposure to NO_2_ contributed to heavy mortality burden in China. Targeted policies reducing the emission of nitrogen oxides and increasing public awareness of the adverse health effects by NO_2_ pollution should be strengthened in China.

## Conflict of Interest

The authors declare no conflicts of interest relevant to this study.

## Supporting information

Supporting Information S1

## Data Availability

The data used in this study were all downloaded from publicly available sources. The national wide NO_2_ monitoring data are available from Zenodo (K. Huang & Liu, 2023), and the corresponding link is https://doi.org/10.5281/zenodo.7869647. The satellite retrieved OMI NO_2_ data are available at https://acdisc.gesdisc.eosdis.nasa.gov/data/Aura_OMI_Level3/OMNO2d.003/. The meteorological data are available at https://www.ecmwf.int/en/forecasts/dataset/ecmwf-reanalysis-v5. The land use data are available at https://vest.agrisemantics.org/content/land-cover-cci-product-user-guide. The annual population and mortality data for each province in China are available at http://www.stats.gov.cn/sj/ndsj/. The Copernicus Atmosphere Monitoring Service (CAMS) reanalysis data are available at https://ads.atmosphere.copernicus.eu/cdsapp#!/dataset/cams-global-reanalysis-eac4?tab=form, and users need to register to access the data.
