# Supplementary material for: Satellite‐Based Long‐Term Spatiotemporal Trends in Ambient NO2 Concentrations and Attributable Health Burdens in China From 2005 to 2020
Source: Geohealth. 2023 May 17;7(5):e2023GH000798. doi: 10.1029/2023GH000798 (PMC10190124; doi:10.1029/2023GH000798)
Supplement: Supplementary file 1 — Supporting Information S1 [file GH2-7-e2023GH000798-s001.pdf]

AGU PUBLICATIONS

GeoHealth

Supporting Information for

Satellite-based long-term spatiotemporal trends in ambient NO_2 concentrations and attributable health burdens in China from 2005 to 2020

Keyong Huang[1,2], Qingyang Zhu[3], Xiangfeng Lu[1,2*], Dongfeng Gu[1,2,4], Yang Liu[3*]

[1]Department of Epidemiology, Fuwai Hospital, National Center for Cardiovascular Diseases, Chinese Academy of Medical Sciences and Peking Union Medical College, 167 Beilishi Road, Xicheng District, Beijing 100037, China

[2]Key Laboratory of Cardiovascular Epidemiology, Chinese Academy of Medical Sciences, Beijing, China

[3]Gangarosa Department of Environmental Health, Rollins School of Public Health, Emory University, Atlanta, Georgia, USA

[4]School of Medicine, Southern University of Science and Technology, Shenzhen 518055, China

***Corresponding author:**

Yang Liu, PhD

Professor and Chair

Gangarosa Department of Environmental Health, Rollins School of Public Health,

Emory University, 1518 Clifton Rd., Atlanta, GA 30322 USA.

Email: yang.liu@emory.edu

Xiangfeng Lu, PhD

Department of Epidemiology, Fuwai Hospital, National Center for Cardiovascular Diseases, Chinese Academy of Medical Sciences and Peking Union Medical College, 167 Beilishi Road, Xicheng District, Beijing 100037, China

Email: xiangfenglu@sina.com

Contents of this file

Fig S1 to S3

Table S1 to S2

Introduction

This supporting information includes the spatial pattern of OMI NO_2 coverage, the spatial distribution of OMI NO_2 before and after imputation, the monthly population-weighted NO_2 levels from 2016 to 2020 in Wuhan and China, the annual monitoring NO_2 level, and the annual coverage of OMI NO_2.

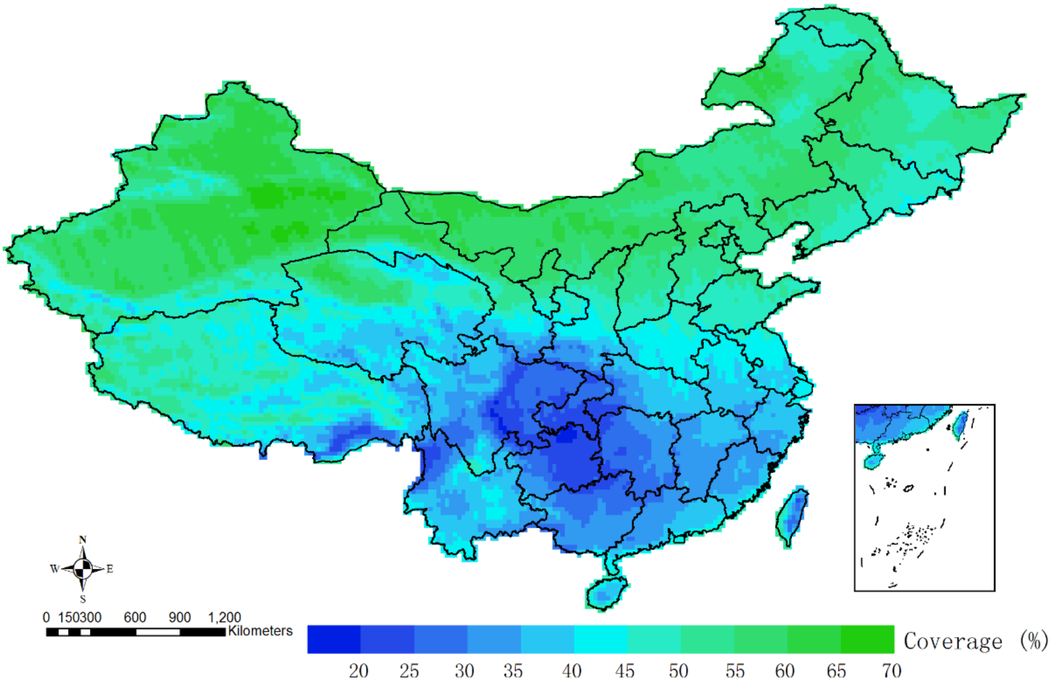

Fig S1. Coverage of OMI vertical column density of NO_2 from 2005 to 2019.

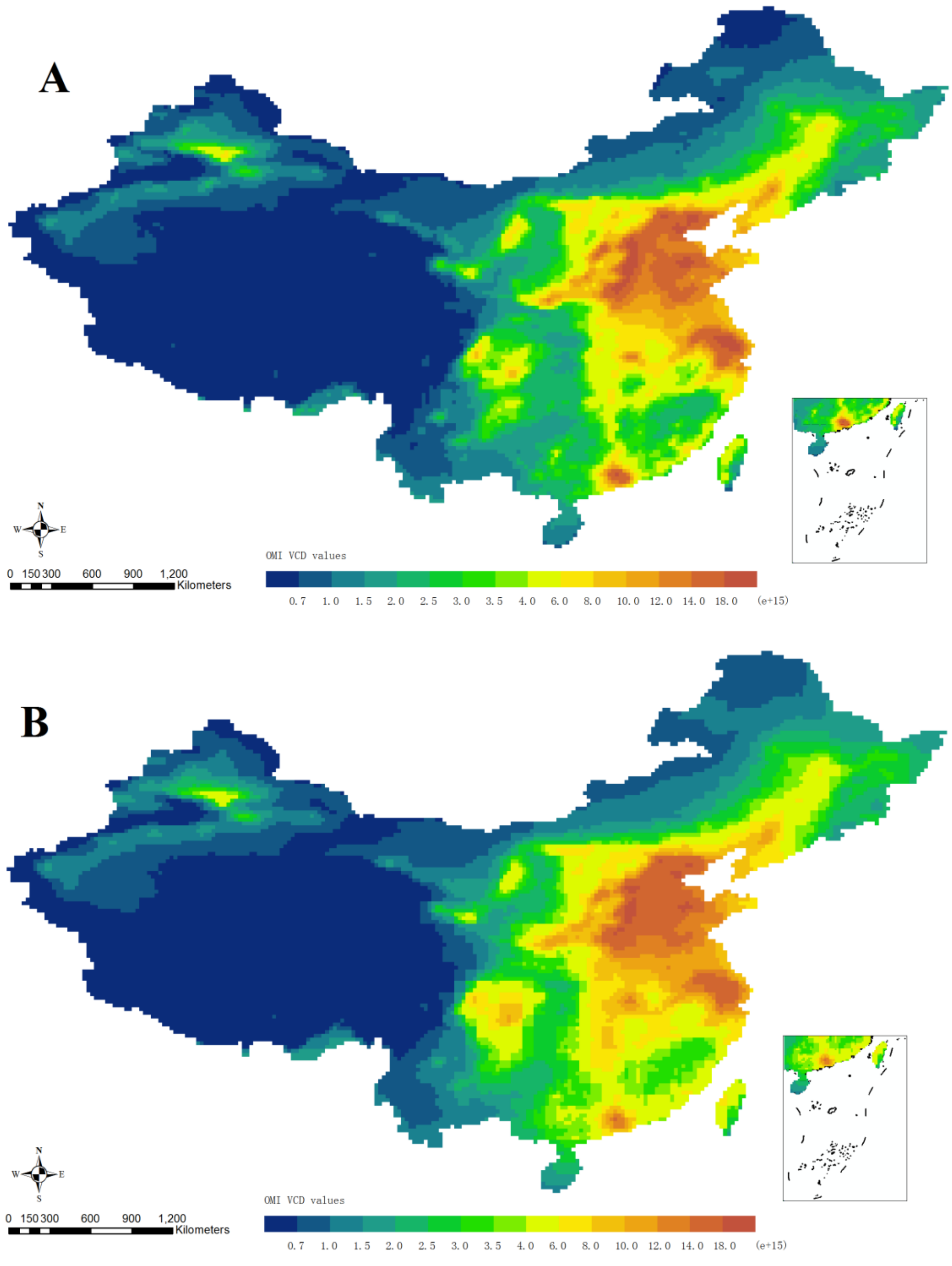

Fig S2. Spatial distribution of average OMI NO_2 vertical column density from 2005 to 2019 in China before imputation (A) and after imputation (B).

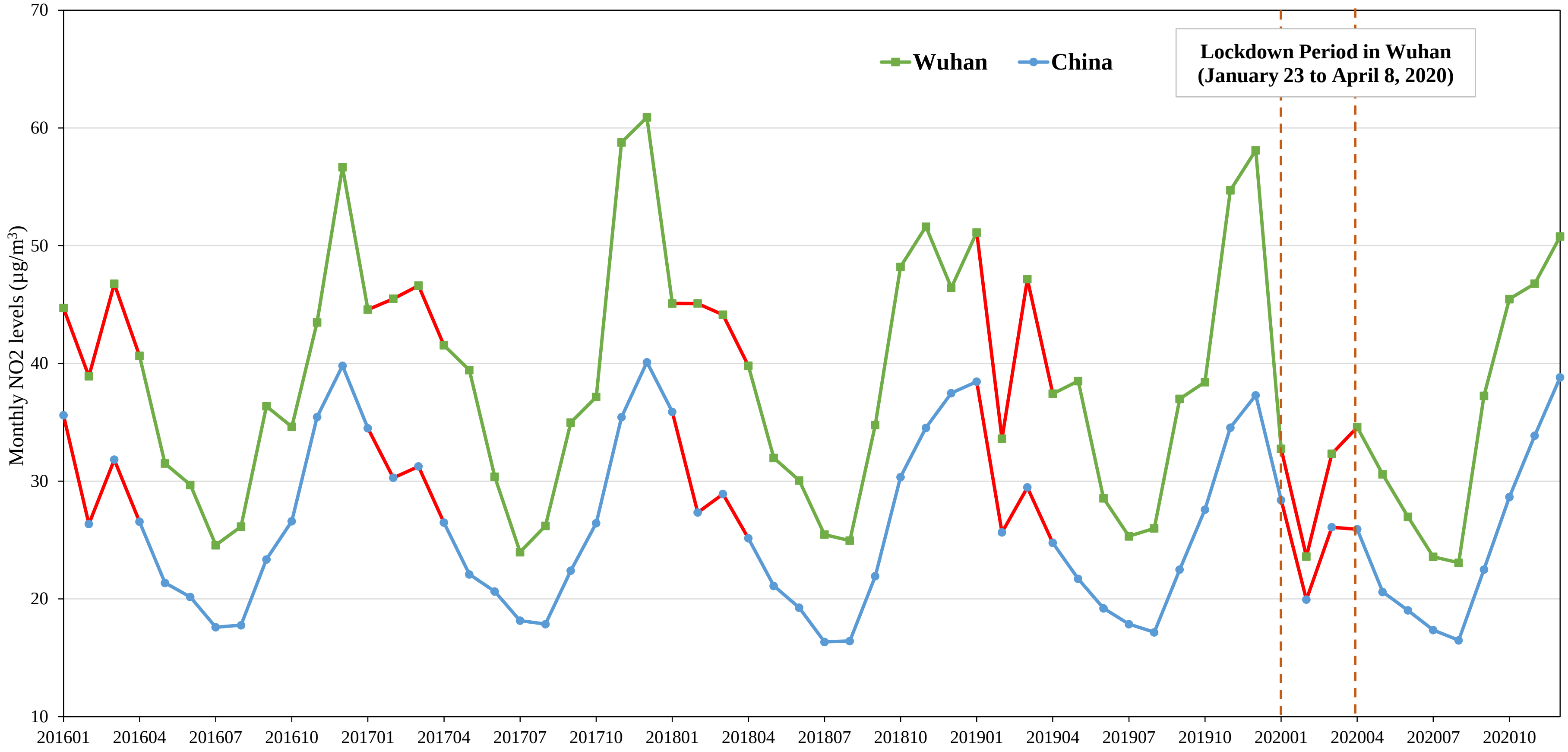

Fig S3. The monthly population-weighted NO_2 levels from 2016 to 2020 in Wuhan and China. NO_2 levels during January to April were highlighted in red.

Table S1. The annual mean and standard deviation of ground NO_2 levels in China.

year	mean (μg/m^3)	standard deviation (μg/m^3)
2014	36.81	16.62
2015	29.89	14.78
2016	29.97	14.91
2017	30.74	14.47
2018	29.03	13.68
2019	29.42	13.69

Table S2. The coverage rate of OMI VCD in China from 2005 to 2019.

Year	Before imputation		After imputation	
	Coverage	Annual mean	Coverage	Annual mean
2005	61.7%	2.270804e+15	100%	2.390e+15
2006	61.7%	2.290928e+15	100%	2.499e+15
2007	62.6%	2.599586e+15	100%	2.795e+15
2008	58.1%	2.529469e+15	100%	2.698e+15
2009	44.8%	2.567642e+15	100%	2.907e+15
2010	39.1%	2.786861e+15	100%	3.198e+15
2011	39.4%	2.997983e+15	100%	3.466e+15
2012	44.9%	2.878061e+15	100%	3.410e+15
2013	45.7%	2.936552e+15	100%	3.269e+15
2014	43.1%	2.670104e+15	100%	3.003e+15
2015	40.8%	2.364919e+15	100%	2.801e+15
2016	39.9%	2.42222e+15	100%	2.835e+15
2017	40.5%	2.491837e+15	100%	2.817e+15
2018	39.9%	2.389335e+15	100%	2.792e+15
2019	40.5%	2.407696e+15	100%	2.769e+15
Total	46.9%	2.559948e+15	100%	2.910e+15
